# *Cyberlindnera jadinii* and *Kluyveromyces lactis*, two fungi used in food processes, have potential probiotic effects on gut inflammation

**DOI:** 10.1128/msystems.00841-23

**Published:** 2023-10-26

**Authors:** Cindy Hugot, Maxime Poirier, Madeleine Spatz, Gregory Da Costa, Chloé Michaudel, Alexia Lapiere, Camille Danne, Valérie Martin, Philippe Langella, Harry Sokol, Marie-Laure Michel, Patrick Boyaval, Mathias L. Richard

**Affiliations:** 1 Université Paris-Saclay, INRAE, AgroParisTech, Micalis Institute, Jouy-en-Josas, France; 2 Paris Center for Microbiome Medicine, Fédération Hospitalo-Universitaire, Paris, France; 3 International Flavors and Fragrances, Neuilly-sur-Seine, France; 4 Gastroenterology Department, INSERM, Centre de Recherche Saint-Antoine, CRSA, AP-HP, Saint Antoine Hospital, Sorbonne Université, Paris, France; Istanbul Medipol University School of Medicine, Istanbul, Turkey

**Keywords:** yeasts, gut microbiota, inflammatory bowel disease

## Abstract

Many strains have been used and selected by the food industry for their capacities to ferment, produce flavors, or produce heterologous molecules. Very little is known about the diversity of foodborne yeasts and their potential effect on gut microbiota and gut health. We initiated a complete characterization of five strains belonging to five species with a long history of safe use in food: *Cyberlindnera jadinii*, *Debaryomyces hansenii*, *Kazachstania unispora*, *Kluyveromyces lactis*, and *Pichia membranifaciens*, with a focus on their capacity to protect against gut inflammation using an *in vivo* dextran sodium sulfate-induced colitis model in mice. *C. jadinii* and *K. lactis* living cells showed a clear reduction in mouse sensitivity to colitis *in vivo*. Interestingly, we observed that *C. jadinii* had the capacity to survive transit in the gut, while *K. lactis* did not. We demonstrated that *C. jadinii* was unable to efficiently adhere to epithelial cells and did not survive more than 24 to 48 h in the gut. Transcriptomic analysis using NanoString technology suggested a potential role of IL-8 through Mif and Fkbp5 in the effect of *C. jadinii* on the immune system. Bacterial and fungal microbiota characterization showed a modification of both microbiota after *C. jadinii* treatment, with a significant increase in positive microorganisms and a decrease in pathobionts. Altogether, these data suggest that both *C. jadinii* and *K. lactis* strains have potential as probiotic yeast strains to fight against inflammation in the gut, but further studies are needed to understand the mechanisms by which these strains act on gut health.

**IMPORTANCE:**

The food industry has always used many strains of microorganisms including fungi in their production processes. These strains have been widely characterized for their biotechnological value, but we still know very little about their interaction capacities with the host at a time when the intestinal microbiota is at the center of many pathologies. In this study, we characterized five yeast strains from food production which allowed us to identify two new strains with high probiotic potential and beneficial effects in a model of intestinal inflammation.

## INTRODUCTION

For several centuries, the production of wine, bread, cheese, and multiple types of fermented food from Europe to Asia relies on the use of fungal strains (1). Over time, academic laboratories and many private companies have fostered research on the diversity of these different yeast strains and their potential in food production through fermentation and aroma production. The repertoire of these strains is now very large and offers a considerable quantity of physiological properties that can be applied in various food processes. Among fungi, the most well-known fungus is the yeast *Saccharomyces cerevisiae* used in wine, beer, and bread production (2). According to G. Khachatourians (2), eight different genera are used today in food biotechnology: *Candida*, *Debaryomyces*, *Kluyveromyces*, *Pachysolen*, *Phaffia*, *Pichia*, *Saccharomyces*, and *Yarrowia*. However, each company has a specific collection of strains for various purposes, from beverage to ethnic food or even food pigment production. These fungal strains can be used as biochemical factories for bioproduct production but are also used alive in food products as they are in cheese. As such, these yeast strains are ingested alive and can accumulate in the digestive tract. While they are considered innocuous as they have been used for decades in food production for humans, very little is known about their potential positive effect on the host. Indeed, these fungi have been associated with food processes by traditional or biotechnological studies but with a focus on either aroma production or fermenting potentials, for instance. Whether these strains can have beneficial effects directly on the host or indirectly by modification of the fungal-bacterial microbiota equilibrium is unknown and seldom studied. In this work, we comprehensively characterized five yeast strains used in food processes to define their capacities to show anti-inflammatory properties and persist in the gut: *Cyberlindnera jadinii*, *Debaryomyces hansenii*, *Kazachstania unispora*, *Kluyveromyces lactis*, and *Pichia membranifaciens*. All these strains have been chosen because they are largely used in the food industries and are likely to be present in relatively high quantities in the daily diet. They are very common strains used in well-known cheese or meat productions, especially in France and Italy, for their capacities to develop cheese aroma and to modify the ambient pH or for the cheese crust production. *D. hansenii*, *P. membranifaciens*, *K. lactis*, and *C. jadinii* are mostly used for cheese crust production and cheese ripening of the Camembert, Brie, Munster, or Pecorino (3–5). While *K. unispora* is part of small mixed microbial consortia used in traditional dairy products like the kefir, it has also been described to be part of the sourdough fermentation processes (6, 7). Interestingly, *D. hansenii* in addition to its role in cheese aroma production has been described in the microbiota developed at the surface of several food products and is used as biocontrol agent against other micro-organisms like *Mucor* or other contaminating molds (8). This work was performed using *in vitro* assays and an *in vivo* mouse model of inflammation on these five yeasts strains. All these strains are used in different steps of food production for aroma or protective microbiota, and despite a long history of safe use for animal or human foods, there are little to no data on their potential effect on gut health.

## MATERIALS AND METHODS

### Cell culture

Human enterocyte-like Caco-2 (ATCC, Virginia, USA) cells were grown in Dulbecco’s modified Eagle’s medium (DMEM) (GIBCO Fischer-Scientific, France) supplemented with 4.5 g/L D-glucose (Sigma-Aldrich, France), pyruvate (Sigma-Aldrich, France), 10% heat-inactivated fetal calf serum (FBS, Eurobio Scientific), 1% nonessential amino acids (GIBCO Fischer-Scientific, France), 1% L-glutamine (Sigma-Aldrich, France), 50 IU/mL penicillin (Sigma-Aldrich, France), and 50 µg/mL streptomycin (Gibco, Fischer-Scientific France) at 37°C in a 5% CO_2_ atmosphere. The growth medium was changed every day with fresh medium and without the addition of antibiotics for the last 24 h of incubation prior to performing the adhesion assays.

HT29-MTX (Micalis Institut) from the human colon was grown in DMEM supplemented with 4.5 g/L D-glucose (Sigma-Aldrich, France), pyruvate (Sigma-Aldrich, France), 10% heat-inactivated (Eurobio Scientific), 50 IU/mL penicillin (Sigma-Aldrich, France), and 50 ug/mL streptomycin (Gibco Fischer-Scientific France) at 37°C in a 5% CO_2_ atmosphere. The growth medium was changed every day with fresh medium and without the addition of antibiotics for the last 24 h of incubation prior to performing the adhesion assays.

The HT-29 cell line (passages P3 to P10) was obtained from the supplier European Collection of Authenticated Cell Cultures. HT29 cells were cultured in DMEM containing 200 mM glutamine (Gibco, Fischer-Scientific, France), 50 IU/mL penicillin (Sigma-Aldrich, France), 50 µg/mL streptomycin (Gibco, Fischer-Scientific, France), and 10% heat inactivated FBS (Eurobio Scientific) at 37°C in a 5% CO_2_ atmosphere. The growth medium was changed every 2 days with fresh medium. At a confluence of approximately 80%, HT29 cells were treated with trypsin-EDTA (Gibco Fischer-Scientific, France) and distributed in 24-well plates (Dutscher, France) with 50,000 cells/well for the following 7 days.

### Preparation of fungi

All strains were supplied by the company DuPont Nutrition and Biosciences. *Debaryomyces hansenii* DGCC3541 (*D. han*), *Kluyveromyces lactis* DGCC3550 (*K. lac*), *Cyberlindnera jadinii* DGCC3540 (*C. jad*), *Kazachstania unispora* DGCC11384 (*K. uni*), and *Pichia membranifaciens* DGCC3547 (*P. mem*) were cultivated on yeast extract peptone dextrose (YEPD) agar (Sigma-Aldrich, France) for 24 h to 48 h at 30°C. The preculture and culture of yeast were prepared in YEPD medium (GIBCO, Fischer-Scientific, France) and then incubated for 24 h at 30°C under agitation at 150 rpm. For the experiments with the culture supernatant, a 24-h culture in YEPD medium (Gibco, Fischer-Scientific, France) was filtered using 0.22 µM filters (VWR International, USA) in order to remove any microorganism and was then used for oral gavage (100 µL per mice) without any other treatment. For heat-killed experiments, the yeasts were culture for 19 h, filtered, and pelleted then subjected to 45 min at 65°C in a water bath. Cells were then wash in phosphate-buffered saline (PBS) and resuspend into the correct concentration for oral gavage. The efficiency of the killing was verified by plating a sample on YEPD medium (Gibco, Fischer-Scientific, France).

### Fungal adhesion on Caco2 and HT29-MTX cells

Caco-2 and HT29-MTX cells were seeded at 5.10^4^ cells per well in 24-well plates (for yeasts) and incubated at 37°C in a 5% CO_2_ atmosphere. After 2 days of culture, the medium was changed every other day until coincubation. Cells were ready to use 15–21 days after confluence for Caco-2 cells and 15–18 days after confluence for HT29-MTX cells when they started to produce mucus. For coincubation, 10^4^ CFU of the yeast strains or 2.10^6^ spores were used per well. Independent experiments were repeated three to six times depending on the sample tested.

#### Quantitative assessment of adhesion

Yeast strain adherence to cultured enterocytes was assayed by the CFU method. After 1 h of coincubation, enterocytes were washed five times with 0.5 mL of PBS (Gibco, Fischer-Scientific, France), lysed with 0.5 mL of PBS (Gibco, Fischer-Scientific, France) with 0.1% Triton X-100 (Sigma-Aldrich, France), scraped if necessary, and collected for CFU determination after serial dilution on YEPD agar plates (yeast culture medium) (Gibco, Fischer-Scientific, France). The adhesion percentage was determined by calculating the ratio between the CFU determined in the inoculum and in the lysate.

#### DNA extraction

Samples were disrupted by using Precellys (Bertin Instruments, USA) with 2 cycles of 20 s at 10,000 rpm. Fifty microliters of 10% SDS were added to the tube, and the tubes were mixed by vortexing and incubated for 30 min at 65°C. After incubation, the samples were disrupted a second time with 1 cycle of 20 s at 10,000 rpm. Then, 200 µL of 5 M K-acetate (Sigma-Aldrich, France) was added to the sample, gently mixed, and incubated overnight at 4°C. After overnight incubation, the samples were centrifuged for 10 min at maximum speed (14,000 × *g*), and the supernatants were transferred to a new 2-mL tube containing 700 µL of isopropanol (Sigma-Aldrich, France). Tubes were mixed gently and incubated at room temperature until DNA precipitation and then centrifuged for 3 min at 8,000 *× g*. The supernatants were discarded, and the pellets were resuspended in 400 µL of H_2_O. Forty microliters of Na acetate 2.5 M, pH 5.2 (Sigma-Aldrich, France), and 1 mL of 100% Et-OH (Sigma-Aldrich, France) were added, gently mixed, incubated at room temperature until DNA precipitation, and centrifuged for 3 min at 8,000 × *g*. The pellets were washed by adding 1 mL of 70% Et-OH and centrifuged for 5 min at 14,000 × *g*. The pellets were dried in a Speed-Vac Dried apparatus (Savant, Thermo Fisher, France). DNA pellets were resuspended in 100 µL of RNAase-free H_2_O overnight at 4°C or 10 min at 50°C, treated with RNase (final concentration, 100 µg/mL; Invivogen, France) and incubated for 15 min at 37°C.

The quality and concentration of DNA were checked using a NanoDrop apparatus (Thermo Fisher Scientific, Massachusetts, USA). Real-time quantitative PCR (RT-qPCR) was performed with 1.5 µg of DNA and using a Luna Universal qPCR Master Mix (New England Biolabs, Massachusetts, USA) in a StepOnePlus apparatus (Applied Biosystems, Foster City, CA, USA) with specific fungal oligonucleotides (TEF1a). Previously, oligonucleotide efficiency was calculated for each strain and used with the charge control sample Ct (adhesion 100%) to determine the sample adhesion percentage.

### IL-8 production by HT29 cells after coculture with fungi

As part of the stimulation protocol, before HT29 stimulation, the cells were stressed for 24 h in medium containing only 5% FBS and not 10%. For stimulation, TNF-α (catalog no. 30001A, Peprotech, France) was added at 5 ng/mL. Coincubation with fungi was realized by adding a yeast suspension with a multiplicity of infection (MOI) of 5 in triplicate, and the plates were incubated for 6 h at 37°C and 5% CO_2_. Independent experiments were repeated two to four times depending on the sample tested.

After treatment, the culture supernatants were collected for the assay. IL-8 quantification was performed using an IL-8 enzyme-linked immunosorbent assay kit (ELISA kit: ELISA Max Deluxe set catalog no. 431504, BioLegend, France) and following the protocol of the provider. Absorbance was measured at 450 nm using a plate spectrophotometer (Tecan Infinite 200Pro, Switzerland).

### Human peripheral blood mononuclear cells (PBMCs) and fungal coincubation

Human PBMCs were purified from whole blood of 27 donors (Etablissement Français du Sang, Le Chesnay, France) using Histopaque-1077 (Sigma Aldrich, France) gradient centrifugation. PBMCs were harvested from the interface, washed three times with sterile PBS, and diluted in 5 mL (for 10 mL of blood) of RPMI 1640 containing 10% FBS (Eurobio Scientific) and 1% penicillin/streptomycin (Sigma-Aldrich, France). The PBMC concentration was determined by using a Kova slide and adjusted to a final concentration of 1 × 10^6^ /mL.

One hundred microliters at 1 × 10^6^ /mL of freshly isolated PBMCs (1 × 106 /mL) were seeded into 96-well culture plates (U-bottom), and 100 µL of a fungal suspension (5 × 10^6^ /mL) was added to each well (triplicate) for a final ratio of 1 (PMBCs):5 (fungi). Lipopolysaccharide (Invitrogen, France) at a final concentration of 10 ng/mL was used as a positive control treatment. To determine cytokine expression, samples were incubated for 24 h at 37°C and 5% CO_2_, and the supernatant was collected for ELISAs.

### Lamina propria immune cell characterization

#### Cell preparation and stimulation

For isolation of lamina propria immune cells, colons were cut out and open longitudinally. Tissues were washed with cold PBS 1× to remove the fecal and then were cut cross-sectionally into 0.5- to 1-cm-long pieces and then mixed with 5 mL pre-warmed HBSS 1× with 5% fetal calf serum (FCS), EDTA (5 mM, Sigma-Aldrich), and DTT (0.145 mg/mL, Sigma-Aldrich) in the shaker at 37°C for 20 min. The supernatants were discarded, and pellets were washed with cold PBS 1×. The tissue pieces were then transferred to a new tube and digested with collagenase type VIII (0.5 mg/mL, Sigma-Aldrich) supplemented with DNase I (1 mg/mL, Roche) for 30 min. All the contents were passed through a 100-mm cell strainer. Lamina propria lymphocytes (LPLs) were obtained using the 40/80 Percoll centrifugation (GE Healthcare). Cells were centrifuged briefly and suspended in complete RPMI containing 10% (vol/vol) FCS (Eurobio Scientific), 1% HEPES (Gibco), 100 U/mL penicillin, and 100 mg/mL streptomycin (Sigma-Aldrich), and 50 µM 2-mercaptoethanol (Sigma-Aldrich).

For mouse mesenteric lymph node preparation, lymph nodes were homogenized and washed in complete RPMI. Mouse cells were then stimulated in culture medium containing 50 ng/mL PMA (Sigma-Aldrich) and 1 mg/mL ionomycin (Sigma-Aldrich) for 3 h at 37°C, 5% CO2, in the presence of 10 mg/mL Brefeldin-A (Sigma-Aldrich).

#### Flow cytometry

Single-cell suspensions were prepared in FACS buffer: PBS 1× (Gibco) supplemented with 2% (vol/vol) FCS (Eurobio Scientific), 0.01% (vol/vol) sodium azide (Sigma-Aldrich). Cells were stained on ice in PBS 1× (Gibco) with Zombie Aqua Fixable Viability Kit (BioLegend). Cells were surface stained in FACS buffer with the following antibodies from eBioscience: FITC-labeled anti-mouse CD3ε (145-2C11), anti-CD16/32 (93); from BioLegend: BV785-labeled anti-mouse CD4 (RM4-5) and BV605-labeled anti-mouse CD8a (53-6.7). Cells were washed in FACS buffer. In mouse, CD4+ alpha beta T cells are CD3+ CD4+ CD8−.

After surface staining, cells were fixed using 4% (vol/vol) paraformaldehyde (PFA; Electron Microscopy Sciences) in PBS 1×. Cells were permeabilized in FACS buffer supplemented with 0.5% (wt/vol) saponin (Sigma-Aldrich). Intracellular staining was performed in the same permeabilization buffer with the following antibodies from eBioscience: APC/Cy7-labeled anti-IFN-gamma (XMG1.2); from BioLegend: PE-Cy7 anti-mouse IL-10 (JES5-16E3).

Flow cytometry was carried out by using LSR Fortessa X-20 (BD). Data were analyzed by using FlowJo software (BD Biosciences).

### Colitis model in mice

Eight-week-old female C57BL/6J mice were purchased from Janvier Laboratory (Le Genest, France) and used 1 week after reception. Animals were kept in humidity- and temperature-controlled rooms under a 12-h light-dark cycle and had access to a chow diet and water *ad libitum*. All experiments were performed in accordance with the ethics committee “Comite d’Ethique en Experimentation Animale” (COMETHEA C2EA – 45, Jouy en Josas, France) approval APAFIS#1579-2015083118218759. Every experiment was repeated at least two times with five mice, with total *n* = 10.

Prior to DSS administration, the mice were gavaged with a suspension of fungi, 1.10^7^ CFU per gavage/mouse/day. When cyclosporin was mentioned, cyclosporin A (Sigma-Aldrich, France) was administered intraperitoneally as follows: 100 µL per mouse at a concentration of 25 mg/kg, three times per week during the length of the experiments after the beginning of the DSS treatment (9).

One week after starting the fungal administration, mice were given 2% (wt/vol) DSS colitis grade (molecular weight, 36,000–50,000; MP Biomedicals, Solon, OH) dissolved in the drinking water *ad libitum* for 7 days, followed by a recovery period (water only) of 5 days. Animals were monitored daily for weight loss and disease activity index (DAI). The DAI is described in Table S1 and includes three parameters with a score notation from 1 to 4: weight loss, stool consistency, and presence of blood in the feces.

For quantification of yeast in fresh stool, stool samples were collected over the course of the study to determine the quantity of yeast remaining after intragastric gavage. Fresh stool samples were weighed and suspended in PBS (Gibco Fischer-Scientific, France) in a proportion of 40 µL/mg feces. For quantification, the dilutions of feces were plated on YEPD agar (Sigma-Aldrich France) plates supplemented with ampicillin (100 mg/mL Sigma-Aldrich France) and penicillin/streptomycin (50 mg/mL Gibco Fisher-Scientific France) and incubated at 30°C for 24 to 48 h. The fungi were then counted, and the absolute quantities of yeast were determined according to the corresponding dilutions.

### Tissues and samples

Mice were euthanized by cervical dislocation. The distal colon was fixed in 4% paraformaldehyde (Electron Microscopy Sciences, Hatfield, PA, USA), and the proximal colon was flushed and frozen for further RNA extraction. Fecal samples were collected at day 7 and at the end of the protocol (day 12) and frozen for gut microbiota analysis and fecal lipocalin-2 (LCN2) level measurements. All samples were stored at −80°C until use.

### Quantification of fecal LCN2 levels

Frozen fecal samples were weighed and suspended in cold PBS. The samples were then agitated on a Precellys (Bertin Corp., France) for 40 s at 5,000 rpm with 4.5 mm glass beads to obtain a homogenous fecal suspension. The samples were then centrifuged for 5 min at 10,000 × *g* (4°C), and clear supernatants were collected and stored at −20°C until analysis. LCN2 levels were estimated using a DuoSet murine LCN2 ELISA kit (R&D Systems, Minneapolis, USA) according to the manufacturer’s instructions and expressed as pg/mg stool.

### RNA extraction and gene expression analysis using nanoString technology

Total RNA was isolated from colon samples using a RNeasy Mini Kit (Qiagen, Hilden, Germany), including a DNAse treatment step, according to the manufacturer’s instructions. The quality and concentration of RNA were checked using a NanoDrop apparatus (Thermo Fisher Scientific, Massachusetts, USA). NanoString analysis was performed and analyzed according to the manufacturer’s recommendations. The panel used for this analysis was the “Immunology V1” panel from nanoString; it was composed of 561 genes and described the following pathways: adaptative and innate immune system, apoptosis, autophagy, signaling pathways (B-cell receptor, T cell receptor, NH-kappa B, NLR, TGF-beta, TGF-beta, TLR, TNF family, type I and II interferon, chemokine, and cytokine), cell adhesion, complement system, hemostasis, host-pathogen interaction, immunometabolism, inflammasomes, lymphocyte activation, and trafficking, MHC class I and II antigen presentation, oxidative stress, phagocytosis and degradation, TH1, TH2, Treg, and TH17 differentiation, and transcriptional regulation.

### 16S DNA gene and ITS2 sequencing

Bacterial diversity was determined for each sample by targeting a portion of the ribosomal genes. PCR was performed to prepare amplicons using V3-V4 oligonucleotides (PCR1F_460: 5′ CTTTCCCTACACGACGCTCTTCCGATCTACGGRAGGCAGCAG 3′, PCR1R_460: 5′ GGAGTTCAGACGTGTGCTCTTCCGATCTTACCAGGGTATCTAATCCT 3′). Amplicon quality was verified by gel electrophoresis, and samples were sent to the @BRIDGe platform for the sequencing protocol on an Illumina MiSeq (Illumina, San Diego, CA, USA).

A similar approach was used for fungal microbiota using the primers ITS2 (sense) 5′-GTGARTCATCGAATCTTT-3′ and (antisense) 5′-GAT ATGCTTAAGTTCAGCGGGT-3′ and the optimized and standardized ITS2-amplicon-library preparation protocol (Metabiote, GenoScreen).

### 16S and ITS2 sequence analysis

The 16S sequences were demultiplexed and quality filtered using the QIIME version 2.1.0 software package. The sequences were then assigned to OTUs using the UCLUST algorithm with a 97% pairwise identity threshold and classified taxonomically using the SILVA reference database (version 13.8) for bacteria. For the ITS sequences, data were processed using the FROGS pipeline (10) for sequence quality control, and filtering and affiliation of taxa were performed with the UNITE ITS database (version 8_2) (11) using the FROGS guidelines for ITS data (http://frogs.toulouse.inra.fr/). Rarefaction analysis was performed and used to compare the relative abundance of OTUs across samples. Alpha diversity was estimated using the Shannon diversity index or the number of observed species. Beta diversity was measured using the Jaccard distance matrix and was used to build principal coordinates analysis plots. The linear discriminant analysis (LDA) effect size (LEfSe) algorithm was used to identify taxa that were specific to treatment. Deposition of the raw sequence data in the SRA database from the NCBI; the accession numbers are as follows: PRJNA879435.

### Statistical analysis

GraphPad Prism version 7 (San Diego, CA, USA) was used for all analyses and preparation of graphs. For all data displayed in graphs, the results are expressed as the mean ± SEM (*n* = 7 to 23 per group). The D’Agostino and Pearson test of normality was applied to all data sets, and in cases where the data did not demonstrate a normal distribution, nonparametric tests were used to analyze statistical differences. For comparisons between two groups, Student’s *t* test for unpaired data or nonparametric Mann–Whitney test was used. For comparisons between more than two groups, one-way analysis of variance and post hoc Tukey test or nonparametric Kruskal–Wallis test followed by a post hoc Dunn’s test was used. For all statistical tests, differences with a *P* value less than 0.05 were considered statistically significant: *P < 0.05, **P < 0.01, ***P < 0.001. Statistical signiﬁcance of sample grouping for β-diversity analysis was performed using Permanova method (999 permutations).

## RESULTS

### *Kluyveromyces lactis* and *Cyberlindnera jadinii* improve recovery after DSS-induced colitis

**Fig 1:**
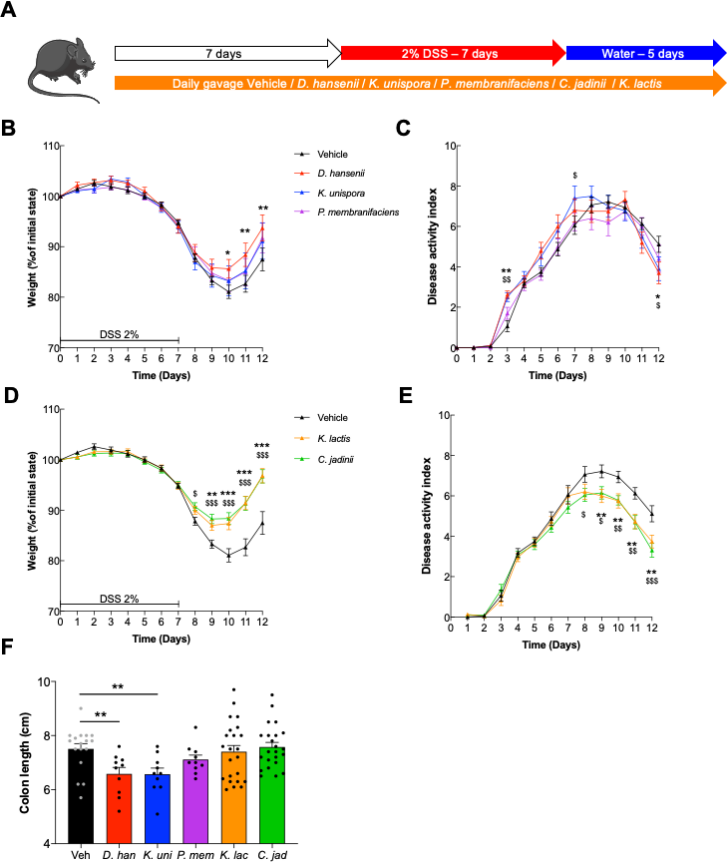
Dextran sodium sulfate-induced colitis is improved with *K. lactis* or *C. jadinii* supplementation. (**A–D**) Mice received *Debaryomyces hansenii* (*D. han*), *Kazachstania unispora* (*K. uni*), *Pichia membranifaciens* (*P. mem*), *Kluyveromyces lactis* (*K. lac*), or *Cyberlindnera jadinii* (*C. jad*) and then 2% DSS for 7 days. Vehicle *n* = 17, *D. han n* = 10, *K. uni n* = 10, *P. mem n* = 10, *K. lac n* = 23, *C. jad n* = 23. A. Experimental design for the administration of DSS. (B and D) Weight of DSS-exposed mice. For statistical comparisons in the upper panel, * indicates *D. hansenii* versus vehicle, and $ indicates *K. unispora* versus vehicle. For statistical comparisons in the lower panel, * indicates *K. lactis* versus vehicle and $ indicates *C. jadinii* versus vehicle. (**C and E**) DAI of DSS-exposed mice. For statistical comparisons in the upper panel, * indicates *D. hansenii* versus vehicle, and $ indicates *K. unispora* versus vehicle. For statistical comparisons in the lower panel, * indicates *K. lactis* versus vehicle, and $ indicates *C. jadinii* versus vehicle. (**F**) Length of the colons of mice treated with DSS. *^,$^*P* < 0.05, **^,$$^*P* < 0.01, ***^,$$$^*P* < 0.001.

Five yeast strains from International Flavors and Fragrances’ (IFF) collection (DGCC), used in food industries for decades, have been selected to test them in a complex model of gut inflammation. The rationale was to directly monitor the potential effects of these strains in a model of inflammation as a screening tool for possible probiotic properties. Some probiotic strains have the capacity to reduce the level of inflammation in this model or to increase the rapidity of recovery. We thus tested the effect of these fungal yeast cells on this model by pretreating the mice with a daily gavage of one strain of yeast for 1 week before beginning the DSS treatment and throughout all DSS administration (Fig. 1A). When treated with 10^7^ yeasts per day of *Kazachstania unispora* or *Pichia membranifaciens*, the mice did not show any protective or worsening effect during DSS treatment while monitoring the weight curves or the symptoms associated with the colitis (less weight loss, diarrhea, and blood in the feces) monitored in the DAI for these two strains (Fig. 1B and C). Nevertheless, mice supplemented with *Debaryomyces hansenii* showed a better recovery from days 10 to 12 for weight loss than the control but not for the other symptoms (Fig. 1B and C).

However, treatment with *Cyberlindnera jadinii* or *Kluyveromyces lactis* strains allowed a significant improvement during the flare period and the recovery phase, as illustrated in Fig. 1D, from day 8 to the end of the experiment. The levels of protection were comparable to the levels reached when cyclosporin A, an anti-inflammatory molecule, was used as a positive control (Fig. S1). Additionally, the symptoms (DAI) were also reduced when the mice were treated with *C. jadinii* or *K. lactis* compared to the control group (Fig. 1E). The measurement of colon length, another macroscopic marker of colitis, did not show any significant improvement (Fig. 1F). Altogether, these results demonstrated that *C. jadinii* and *K. lactis* can have a positive effect on inflammation when given to mice.

### Neither the medium supernatant nor the dead yeast cells have a comparable positive effect on gut inflammation

To identify possible effectors of the two potential probiotics, we tested whether the culture supernatant or the dead cells could elicit comparable protective effects during colitis. Using the same DSS-induced colitis model in mice, both hypotheses were tested. In Fig. S2A and B, pure filtered 24-h spent culture medium of each yeast was given to mice 7 days before and during DSS colitis and compared to fresh medium (Vehicle, YEPD). None of the treatments improved colitis recovery, and in contrast, they had a tendency to worsen the symptoms. For the second experiment, yeast cells were heat killed, washed, and resuspended in PBS before gavage into mice 7 days before and during DSS colitis. Treatment of mice with these samples had no beneficial effect on mouse weight loss independent of the yeast used for the culture (Fig. S2C and D). These results suggest that the two living yeasts are mandatory for the effect on the host and that at least the molecules produced by both yeasts in this rich medium are not directly involved in the effect on colitis recovery.

### The persistence capacities of *K. lactis* and *C. jadinii* in the mouse gut are completely different

**Fig 2:**
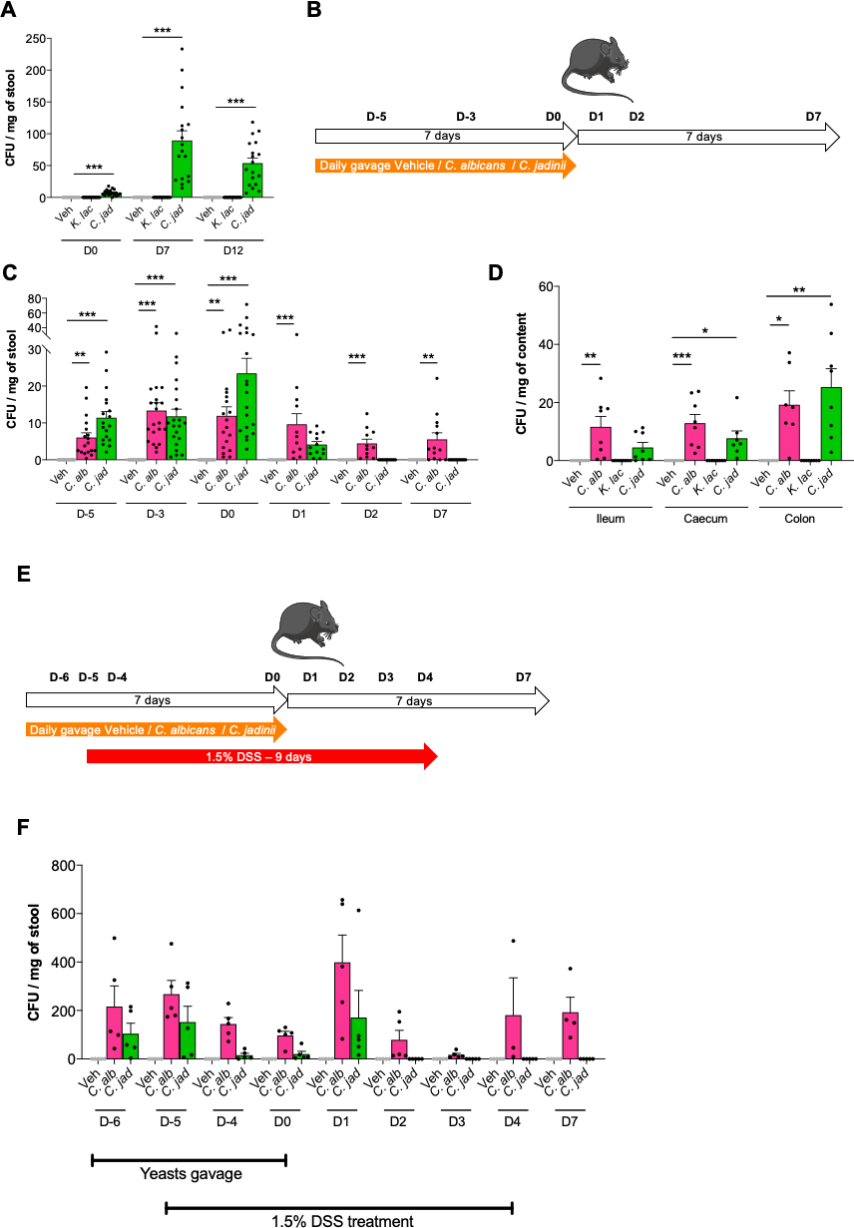
Persistence capacities of *C. jadinii* and *K. lactis* in the mouse gut are completely different but not influenced by inflammatory conditions. (**A**) Mice received *Kluyveromyces lactis* (*K. lac*) or *Cyberlindnera jadinii* (*C. jad*) and then 2% DSS for 7 days (see Fig. 1A). Vehicle *n* = 20, *K. lac n* = 20, *C. jad n* = 20. (**A**) Fungal quantity in feces during DSS treatment, expressed in CFU. (**B–C**) Mice received *Candida albicans* (*C. alb*) or *Cyberlindnera jadinii* (*C. jad*) for 7 days. Vehicle *n* = 13–21, *C. alb n* = 13–21, *C. jad n* = 13–21. (B) Experimental design for the administration of yeasts. (**C**) Fungal quantity in feces during and after yeast supplementation treatment, expressed in CFU. (**D**) Mice received *Candida albicans* (*C. alb*), *Kluyveromyces lactis* (*K. lac*), or *Cyberlindnera jadinii* (*C. jad*) for 7 days. Vehicle *n* = 8, *K. lac n* = 8, *C. jad n* = 8. (**D**) Fungal quantity in the ileum, cecum, and colon after 7 days, expressed in CFU. (**E–F**) Mice received *Kluyveromyces lactis* (*K. lac*), *Cyberlindnera jadinii* (*C. jad*), or the vehicle for 7 days and then 1.5% DSS for 9 days. Vehicle *n* = 5, *K. lac n* = 5, *C. jad n* = 5. F. Fungal quantity in feces before, during, and after DSS treatment, expressed in CFU. For statistical comparisons, * indicates versus the appropriate vehicle. *P < 0.05, **P < 0.01, ***P < 0.001.

Since live cells are necessary for the effect on inflammation, we monitored the level of living yeast present in the feces during DSS-induced colitis. This experiment showed that no live *K. lactis* were found at any time point before or after colitis, while *C. jadinii* cells seemed to persist or even colonize the gut tract (Fig. 2A).

To determine how long *C. jadinii* can persist after the last oral gavage in the gut and if the persistence was different along the gastrointestinal tract, we monitored the CFU of *C. jadinii* regularly during and after stopping a week of oral gavage (Fig. 2B). The yeast strain *Candida albicans* was added to this experiment as a positive control of persistence. After 2, 5, or 7 days of oral gavage of 10^7^ cells/mouse, *C. albicans* and *C. jadinii* were both viable in the feces (Fig. 2C). After cessation of the gavage, although the global level of *C. albicans* decreased, there was still a good abundance of *C. albicans* cells in the feces of the mice, while the levels of *C. jadinii* decreased very rapidly at day 1 and became undetectable at day 2 (Fig. 2C). These data suggest that *C. jadinii* resists transit through the entire gut after oral gavage but does not persist in the gut or colonize it. We also tested whether oral gavage with the cell suspension had any effect on mouse weight gain during the experiment, but we did not detect any significant effect of any strain on mouse weight (data not shown).

While we have shown that only *C. jadinii* survived the gut environment after oral gavage, we did not know whether *K. lactis* can resist in the gut after ingestion and where in the gut. To test this hypothesis, we set up an *in vivo* experiment during which we monitored the level of the colonization of the two yeasts in different sections of the gut after 1 week of oral gavage. Again, *C. albicans* was added to this experiment as a positive control. Both *C. albicans* and *C. jadinii* persist in the ileum, the cecum, and the colon, while *K. lactis* did not survive even in the ileum (Fig. 2D).

As *C. jadinii* cell load measured during the DSS treatment (Fig. 2A) seemed to be a little superior to the levels detected during the persistence test (Fig. 2C), we hypothesized that the inflammatory conditions might have been conditions favoring the persistence and the growth of *C. jadinii*. To test this hypothesis, an experiment *in vivo* of daily yeast gavage, while under 1.5% DSS treatment to induce a low inflammation, was performed, and we followed the persistence of *C. jadinii* and *C. albicans* in these settings (Fig. 2E). However, even in these gut inflammatory conditions, the level of *C. jadinii* persistence is not modified, since after 2 days no *C*. *jadinii* live cells can be detected (Fig. 2F).

### The persistence of *C. jadinii* in the gut is not explained by better adhesion abilities

**Fig 3:**
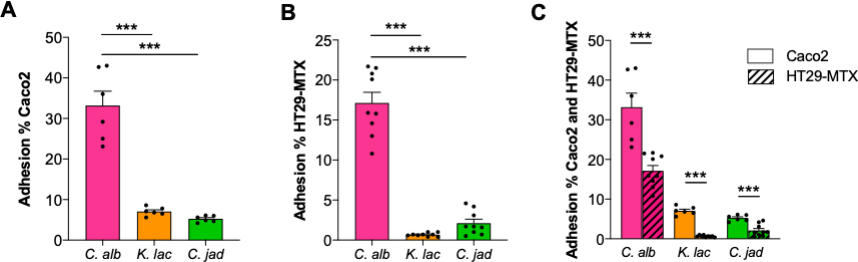
*K. lactis* and *C. jadinii* have the same adhesion abilities. (**A–C**) Adherence capacity of *Candida albicans* (*C. alb*), *Kluyveromyces lactis* (*K. lac*), and *Cyberlindnera jadinii* (*C. jad*) on (**A**) Caco-2, (**B**) HT29-MTX, and (**C**) both cell lines. For statistical comparisons, * indicates versus *C. albicans*. ***P < 0.001.

As a large quantity of live *C. jadinii* cells survive in the gut after DSS treatment, we suggest a possible ability of *C. jadinii* cells to adhere to and survive in the mouse gut. Additionally, how *K. lactis* interacted with the epithelial cells was also important to monitor, even if no live cells were detected in the feces. Hence, to test whether these yeasts were able to adhere to epithelial cells, we performed an adhesion assay *in vitro* on two types of cells: Caco2 and HT29-MTX cells. Both are human cells with different properties: Caco2 are absorptive epithelial cells, while HT29-MTX are mucus secretive cells. Comparing these two types of cells would provide details on how mucus may affect fungus-to-cell adhesion. In Fig. 3, we observed that the capacity of adhesion on Caco2 cells of both *K. lactis* and *C. jadinii* strains was near 10% (Fig. 3A) and less than 5% on HT29-MTX (Fig. 3B). Nevertheless, we observed by comparison with *C. albicans*, a positive control for epithelial cell adhesion, which neither *K. lactis* nor *C. jadinii* had strong adherence abilities. However, mucus seems to decrease the adhesion for all strains, since they adhered less to HT29-MTX cells than to Caco2 cells (Fig. 3C). A test for the modification of the adherence in inflammatory conditions with HT29 after TNF-alpha induction (see “IL-8 production by HT29” in the Materials and Methods section for the experimental conditions) was also performed but did not demonstrate any modification of adhesion of any strains (data not shown). These results suggest that the persistence of *C. jadinii* in the mouse gut is not due to colonization of the gut surface.

At this point of the study, no mechanistic explanations could be proposed to elucidate the protective effects of both strains. One possible explanation was a modulation of the host response by the yeast cells. To explore this, we performed several *in vitro* experiments with epithelial or immune cells as well as targeted quantification of host markers using nanoString technology.

### *C. jadinii* yeast cells can reduce IL-8 production in TNF-α-treated HT29 epithelial cells

**Fig 4:**
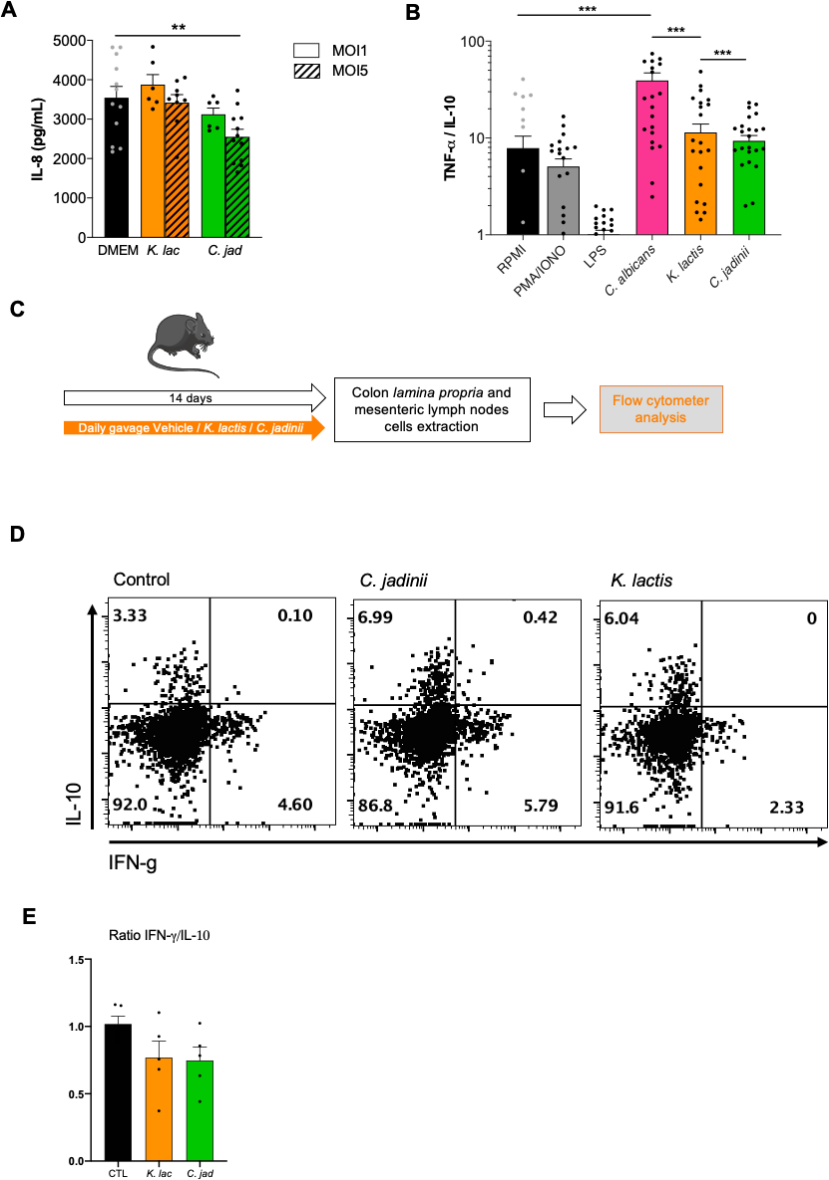
*C. jadinii* yeast cells can reduce IL-8 production in TNF-α-treated HT29 epithelial cells. (**A**) IL-8 production by HT29 after coculture with *Kluyveromyces lactis* (*K. lac*) or *Cyberlindnera jadinii* (*C. jad*) strains at an MOI of 1 and an MOI of 5, with TNF-α priming. For statistical comparisons, * indicates versus DMEM. **P < 0.05. (**B**) Ratio of TNF-α/IL-10 production of the culture supernatants of PBMCs co-incubated with *Candida albicans* (*C. alb*), *Kluyveromyces lactis* (*K. lac*), or *Cyberlindnera jadinii* (*C. jad*) for 24 h at an MOI of 5. (**C–E**) Mice received *Kluyveromyces lactis* (*K. lac*), *Cyberlindnera jadinii* (*C. jad*), or the vehicle for 14 days, and colon lamina propria and mesenteric lymph nodes cells were collected. Vehicle *n* = 5, *K. lac n* = 5, and *C. jad n* = 5. (**D–E**). Representative plots of gated CD4 T cells from colon lamina propria from mice treated with PBS (control), *C. jadinii*, or *K. lactis*. (**D**) Intracellular analysis of IL-10 and IFN-g expression by gated CD4 T cells from colon lamina propria. For all plots, numbers indicate percentage of cells in relevant quadrant. (**E**) IFN-g/IL-10 percentage ratio representation. For statistical comparisons, * indicates versus RPMI. ***P < 0.001.

As surface cells, HT29 epithelial cells have the capacity to produce different types of cytokines to communicate with immune cells at the periphery and regulate the immune system response of the host. One classical proinflammatory molecule produced by HT29 cells is IL-8, which is produced after TNF-α induction. When coincubated individually with the two yeast strains with TNF-α-treated HT29 cells for 6 h, we showed that no significant difference could be observed at an MOI of 1. Nevertheless, at an MOI of 5, *C. jadinii* significantly reduced the amount of IL-8 produced by HT29 cells (Fig. 4A). This finding suggests that part of the effect of *C. jadinii* on gut inflammation might be driven by a reduction in the inflammatory signal triggered by epithelial cells.

### *K. lactis* and *C. jadinii* elicit low levels of inflammatory responses to the human immune system

Within the gut, microorganisms also interact with immune cells via the cells present in the lamina propria. Defining how the yeast cells are recognized and what the response elicited when they encounter immune cells can also reveal why these yeasts affect DSS-induced colitis. Hence, PBMCs extracted from the blood of healthy subjects were coincubated with yeast cells at an MOI of 5, and the culture supernatant was subjected to ELISA analyses for cytokine quantification. In Fig. 4B, the ratio of TNF-α to IL-10 production illustrates the level of the proinflammatory effect of each strain, with the use of *C. albicans* as a positive control. The results showed that compared to the well-known opportunistic and proinflammatory pathogens *C. albicans*, *C. jadinii*, and *K. lactis* do not trigger either strong pro- or anti-inflammatory responses when they encounter peripheral immune cells.

Since all yeasts’ effects observed were localized in the gastro-intestinal tract, we also wanted to characterize the effect of both yeast on the local immune cells on naive mice without any inflammation. With this aim, we gavaged the mice for 14 days with either the vehicle, *C. jadinii*, or *K. lactis* yeast cells in comparable concentrations with the previous experiments. After 14 days, the mice were sacrificed, and the immune cells from the colon lamina propria and the mesenteric lymph nodes were extracted, labeled and analyzed by flow cytometry (Fig. 4C). When we characterized the ratio IFN-gamma/IL-10-positive cells among T CD4 plus in colon lamina propria cells, we were able to see a slight but not statistical decrease when the mice had received the yeast by oral gavage (Fig. 4D and E).

### Colon cytokine profiles after DSS treatment with *C. jadinii* and *K. lactis*

**Fig 5:**
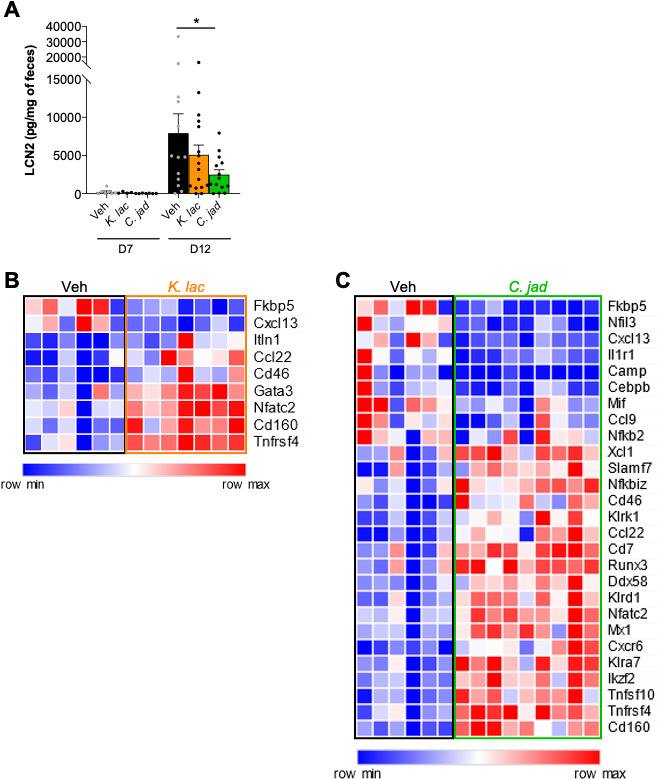
Inflammatory response to *K. lactis* and *C. jadinii* gavage after DSS and 5 days of recovery. (**A–C**) Mice received *Kluyveromyces lactis* (*K. lac*) or *Cyberlindnera jadinii* (*C. jad*) and then DSS for 7 days. (**A**) Intestinal inflammation, expressed as the lipocalin-2 levels in feces at day 7 and day 12. Vehicle *n* = 9–17, *K. lac n* = 7–19, *C. jad n* = 8–17. NanoString on (**B**) *K. lactis* and (**C**) *C. jadinii*. Vehicle *n* = 6, *K. lac n* = 7, and *C. jad n* = 9.

The measurement of lipocalin-2 by ELISA, a marker of gut inflammation present in the feces, showed a clear tendency of reduction with *K. lactis* treatment and was significantly lower than in the control mice after *C. jadinii* treatment at the end of the experiment (Fig. 5A, day 12). Using mRNA extracted from colon samples at day 12 after DSS administration, we investigated whether the inflammatory expression pattern was different compared to that in the control group. nanoString technology was used for very precise quantification of the numbers of ARN molecules present in the samples and targeting immunology-related pathways (cell signaling, innate and adaptative immunity, phagocytosis, etc). We selected the genes significantly modulated with an adjusted *P* value <0.01. Using these parameters, *K. lactis* administration showed a level of genes regulated much lower than with *C. jadinii* treatment: 9 versus 27 (Fig. 5B and C). Both yeasts downregulated FKBP5 and CXCL13 expression at day 12, while *C. jadinii* alone downregulated Nfil3 and Mif. *K. lactis* remarkably triggered the induction of several genes related to the Th2 pathway (GATA3, CCL22, NFATC2, or TNFRSF4), but very few other genes were modulated. In contrast, *C. jadinii* administration induced the overexpression of a larger number of genes: genes from the Th2 pathway were also present with the addition of CD8-related genes (SLAMF7, KLRK1, RUNX3, CXCR6, KLRA7, or CD160). Neither Th2 nor CD8 pathways are commonly linked to gut inflammation, suggesting a global immune system regulation that might be a direct or indirect consequence of *C. jadinii* presence.

To determine whether this global effect was due directly to the presence of *C. jadinii* or indirectly through the modulation of the microbiome or the mycobiome of the mice, we investigated the evolution of both during yeast gavage.

### Microbiota analysis shows a strong impact of *C. jadinii* gavage on the bacterial microbiota

**Fig 6:**
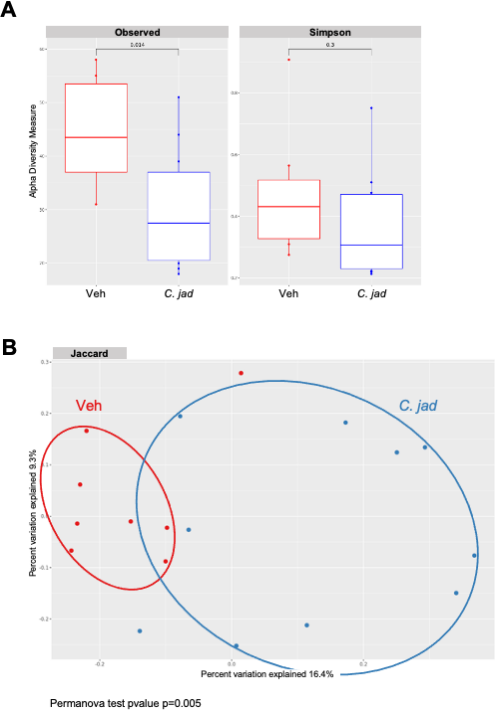
*C. jadinii* administration triggered slight modification of the mycobiota. (**A and B**) Wild-type mice from Janvier Laboratory after 1 week of *Cyberlindnera jadinii* (*C. jad*) oral gavage. (**A**) Observed OTU and Simpson index describing the alpha diversity of the fungal microbiota (ITS2) in the fecal microbiota. (**B**) Beta-diversity. Principal coordinate analysis of Jaccard distance, with each sample colored according to the treatment.

To test whether the effect on inflammation might be indirect and through modulation of the gut microbiota, we performed an amplicon-based analysis of both fungal (ITS2) and bacterial (16S) microbiota of mice after 1 week of gavage with *C. jadinii* or *K. lactis* and just before DSS treatment and compared it to the group receiving the vehicle. Concerning the fungal microbiota (mycobiota), *K. lactis* oral gavage did not modify the fungal alpha- or beta-diversity (data not shown). However, the Simpson diversity index revealed a tendency toward a reduction in diversity after *C. jadinii* oral gavage, while the number of observed OTUs was significantly lower (Fig. 6A). Similar results were observed when comparing the beta diversity of the different groups, with a significant modification of the mycobiota after *C. jadinii* treatment using either Bray‒Curtis (*P* value = 0.005, data not shown) or Jaccard (*P* value = 0.004) indexes (Fig. 6B). While looking for specific taxonomic ranks influenced by the yeast using linear discriminant analysis (LEfSe), we identified seven genera negatively impacted by *C. jadinii*: *Wallemia*, *Vishniacozyma*, *Sporobolomyces*, *Penicillium*, *Sporisorium*, *Oidiodendron*, and *Saccharomyces* (Fig. S3).

**Fig 7:**
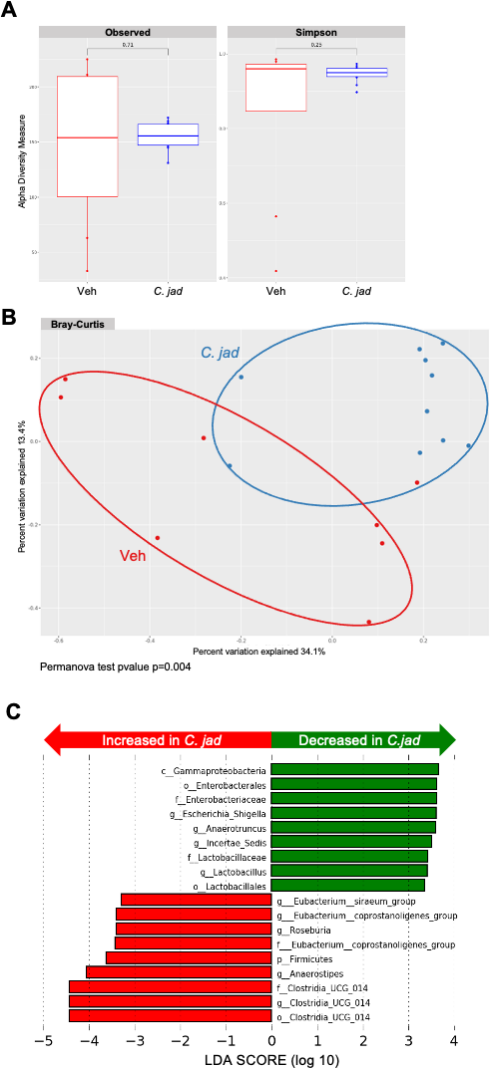
The bacterial microbiota is significantly modified by *C. jadinii* administration. (**A–C**) Wild-type mice from Janvier Laboratory after 1 week of *Cyberlindnera jadinii* (*C. jad*) oral gavage. (**A**) Observed ASF and Simpson index describing the alpha diversity of the bacterial microbiota (16S) in the fecal microbiota. (**B**) Beta-diversity. Principal coordinate analysis of Bray‒Curtis distance, with each sample colored according to the treatment. (**C**) Taxa with the largest differences (LDA >2) in abundance by linear discriminant analysis (LEfSe) (LDA >2).

Bacterial microbiota analysis showed comparable consequences of the gavage of the two yeasts, with no effect of *K. lactis* (data not shown) and a significant modification of the microbiota after *C. jadinii* administration. Global alpha diversity was not modified by *C. jadinii* (Fig. 7A), but the beta-diversity analysis using the Bray‒Curtis index demonstrated a significant effect of the yeast on the bacterial microbiota (*P* value = 0.004) (Fig. 7B). Interestingly, using the LEfSe tool for the identification of discriminant taxa, we observed that *C. jadinii* administration reduced the level of *Enterobacteriaceae*, such as the *Escherichia* and *Shigella* genera, but increased the quantities of Firmicutes, such as the *Anaerostipes*, *Eubacterium*, or *Roseburia* genera (Fig. 7C), a positive remodeling of the bacterial microbiota for inflammatory environments.

## DISCUSSION

Microbiota analysis has reached a considerable level of interest since the 2000s, first through academic research and then quickly in private companies and, more recently, in the public domain. This interest was driven by the discovery of the strong impact the different microbiota can have on human health. The bacterial gut microbiota quickly concentrated the strongest effort due to the higher number of scientists working on bacteria compared to yeasts and to several major discoveries related to human diseases such as diabetes, obesity, and inflammatory bowel diseases (12, 13). Very little interest first was dedicated to the fungal part of the gut microbiota, but with the improvement of the databases and the techniques, the scientific community was able to also link fungal dysbiotic conditions to some human diseases, such as gut inflammation or cancer (14, 15). Interestingly, unlike bacteria, the gut fungal community is largely derived from the food ingested. As such, the fungi used in food processes are central for our comprehension of fungal community modulation and consequently its role.

While the fungi used in food processes are in many cases killed during the process of production, there are foods that are composed of live cells, such as cheeses and some fermented foods or beverages or even some food products that are artificially covered by a chosen microbial community to protect the product from the development of deleterious or pathogenic microbial strains (16). These fungal strains have been used for decades or more in food production than in the food industry and are considered innocuous. However, very little is known about the potential positive effects of these strains on our health, especially now that our techniques of investigation have largely improved and can follow parameters never studied to date. To address this question, we selected five yeast strains from the IFF collection to test their potential probiotic effects on gut health in the context of gut inflammation using *in vitro* and *in vivo* models: *Cyberlindnera jadinii*, *Debaryomyces hansenii*, *Kazachstania unispora*, *Kluyveromyces lactis*, and *Pichia membranifaciens*. The well-described DSS-induced colitis model was used in mice, and two of the five yeast strains, *C. jadinii* and *K. lactis*, demonstrated a significant positive effect in a mouse model of colitis. Further experiments were performed to characterize the capacity of both strains to persist in the gut and to determine how long. We demonstrated that *K. lactis* was very rapidly killed during the transit along the gut, since no live cells were recovered from any part of the small intestine, cecum, or colon. The temperature (37°C) is probably the main cause of this observation, since this yeast growth temperature is around 28°C–30°C, but other environmental parameters can be at play as well (pH, low oxygen levels, etc). On the other hand, *C. jadinii*, as the negative control *Candida albicans,* was able to persist during DSS treatment and in all compartments. However, the persistence of *C. jadinii* was not comparable to that of *C. albicans* after the end of the administration, since live *C. jadinii* cells quickly disappeared and were undetectable after 1 day, while *C. albicans* can persist for several days, similar to *Saccharomyces boulardii* (17). We showed that this persistence was not due to the adherence capacity of *C. jadinii* to mucus or epithelial cells, since the strains harbored adherence capacities comparable to those of *K. lactis* and much lower than those of *C. albicans*. We can hypothesize that *C. jadinii* cells can simply resist the deleterious conditions present during gut transit (low pH, low level of oxygen, 37°C growth conditions) but have no particular features allowing for colonization of the gut.

To understand how both yeast strains affected gut sensitivity to inflammation, we investigated their impact on the host inflammatory response with different experiments. One strategy was to detect the anti-inflammatory potential of the strains using an *in vitro* assay with TNF-α-primed HT-29 human epithelial cells following IL-8 production. Using this assay, we showed that only *C. jadinii* was able to significantly decrease IL-8 production in epithelial cells. Using nanoString technology, to dig into the mechanism potentially at play with *C. jadinii*, we revealed a significant downregulation of the expression of FK506-binding protein 51 (FKBP5), an immunophilin with numerous functions in immunity, hormonal physiology, and protein synthesis (18). Interestingly, it has been shown that Fkbp5, a key molecule in melanoma growth, regulates IL-8 production (19). Whether FKBP5 is expressed in gut cells or immune cells from the lamina propria is not yet clear, but its implication in the induction of proinflammatory molecules indicates a potential new target to follow in further studies. Additionally, the macrophage-inhibition factor (Mif) was also specifically downregulated with *C. jadinii,* and a study identified the role of MIF in inducing the NF-kB pathway, resulting in IL-8 secretion. Binsky et al. (20) described this pathway as a potential target to modulate chronic lymphocytic leukemia. This raises the question of whether *C. jadinii* might also have an effect on melanoma or colon cancer, something that might be interesting to test in the future.

The use of dead cells or the culture supernatant in the same DSS-induced colitis model did not allow us to identify any effectors of this positive effect on gut inflammation, since none of these fractions had a similar effect. Thus, live yeast cells seem mandatory to observe any effect on the mouse physiopathology of gut inflammation in the context of DSS-induced colitis.

To further monitor the effect of the two yeasts on the mice and to investigate the potential mechanism of action, we determined the modification of the bacterial and fungal microbiota after administration of *C. jadinii. C. jadinii* treatment had a wide effect on both bacterial and fungal microbiota. The mycobiota was impacted, with an overrepresentation of *C. jadinii* causing a decrease in many genera compared to the control. No fungi with known effects on inflammatory bowel diseases (IBD) were identified at that step, except for the *Saccharomyces* genus which in these specific conditions were decreased. High abundance of the *Saccharomyces* genus has often been associated with non-IBD samples (21), but in our study, this suggests that the potential indirect positive effect of *C. jadinii* overabundance counteracted the decrease in the *Saccharomyces* genus. However, we cannot rule out the effect of the decrease in abundance in specific fungi that have not yet been investigated for their role in gut inflammation. Interestingly, as previously described, fungi can modulate the bacterial gut microbiota (22), and after *C. jadinii* administration, we did not detect any alpha-diversity modification, but a clear modification of the bacterial beta-diversity and modulation of several bacterial genera was detected. We observed a decrease in *Enterobacteriaceae* with *Escherichia* or *Shigella*, which are well-known bacteria susceptible to initiate or increase inflammation (23). While the presence of *C. jadinii* also caused an increase in genera described for their positive potential in IBD or IBS, such as *Roseburia* or *Anaerostipes*, *Roseburia* has been described with *Faecalibacterium prausnitzii* as decreased in patients with ulcerative colitis, and its supernatant causes amelioration of colitis in mice (24, 25). For Anaerositpes, several publications using microbiota profiling identified that this genus is enriched in healthy subjects in comparison with IBS or IBD patients (21, 26). Such modulation of the bacterial and fungal microbiota of *C. jadinii*-treated mice with an increase in the abundance of positive microorganisms and a decrease in that of deleterious microorganisms suggests that the protective effect observed can be the consequence of these alterations, with direct or indirect effects on host inflammation markers such as IL-8. Such effects suggest that experiments with fecal material transfer or the use of germ-free mice may be used in a future study to investigate whether the modification of the microbiota alone is responsible for the protective phenotype.

This characterization of yeast used in the food industry on their potential probiotic properties allowed for the identification of two strains, *K. lactis* and *C. jadinii*, with improved symptoms when used in a mouse model of colitis. Various tests were carried out to identify the potential mechanism of action of the two yeasts. We were unable to identify a specific action of *K. lactis* in these experiments, but we can speculate that this strain has anti-inflammatory effects on epithelial cells or modifies intestinal permeability, which was not detected by our specific experimental set-up. In the literature, there is no description of such an effect of *K. lactis*, but *K. marxianus* is described in various publications as having anti-inflammatory and anti-oxidative stress properties in *in vitro* and *in vivo* models (27–29).

While we cannot describe the complete mechanism at play yet, we can hypothesize that a direct role on the immune pathway with Fkbp and IL-8 is a possibility as well as the modulation of the bacterial and fungal microbiota that would indirectly affect the host susceptibility to gut inflammation with the enrichment in the *Roseburia* and *Anaerostipes* genera. This study also highlights the importance of characterizing the different strains used in the food industry because they may harbor many properties that are inefficiently used today.

## Data Availability

All data generated or analyzed during this study are included in this published article. The raw sequence data were deposited in the European Nucleotide Archive: PRJNA879435.

## SUPPLEMENTAL MATERIAL

Supplemental material msystems.00841-23-s0001.pdf Table S1 and Figures S1 to S3.

ASM does not own the copyrights to Supplemental Material that may be linked to, or accessed through, an article. The authors have granted ASM a non-exclusive, world-wide license to publish the Supplemental Material files. Please contact the corresponding author directly for reuse.
