# Supplementary material for: Cyberlindnera jadinii and Kluyveromyces lactis, two fungi used in food processes, have potential probiotic effects on gut inflammation
Source: mSystems. 2023 Oct 26;8(6):e00841-23. doi: 10.1128/msystems.00841-23 (PMC10734524; doi:10.1128/msystems.00841-23)
Supplement: Supplemental material — Table S1 and Figures S1 to S3. [file msystems.00841-23-s0001.pdf]

Supplemental Table 1: Disease Activity Index (DAI) description. The DAI describes the level of disease symptoms. Weight loss and feces from mice were monitored daily. For the quotation, three parameters are followed with a score notation from 1 to 4: weight loss, stool consistency and presence of blood in the feces.

Score	Consistency stool	Blood	Weight loss
0	Normal	Negative (-)	<1%
1			1% - 5%
2	Loose	Trace of blood	6% - 10%
3			11% - 20%
4	Diarrhea	Bleeding	> 20%

Supplemental Figure 1

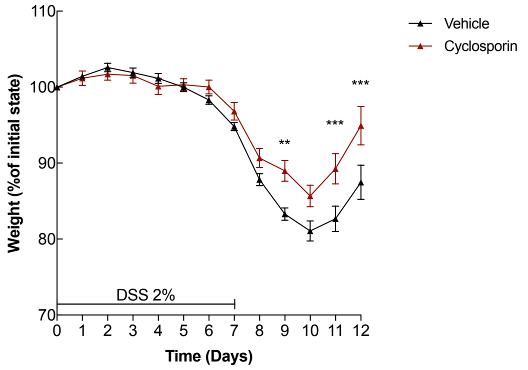

Supplemental Figure 1: Cyclosporine administration effects on gut inflammation in DSS model.

Mice received the vehicle (Vehicle) or Cyclosporin (25 mg/kg) and then dextran sulfate sodium (DSS) for 7 days. Vehicle n = 17, Cyclosporin n = 10. For statistical comparisons **p <0.01, ***p<0.001.

Supplemental Figure 2

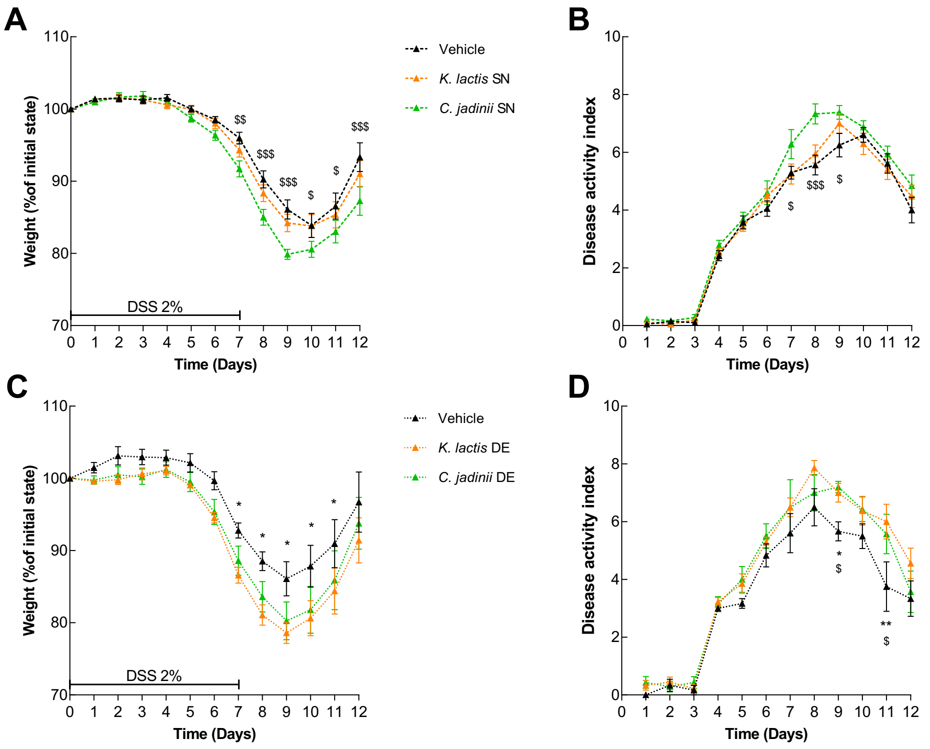

Supplemental Figure 2: Neither the medium supernatant nor the dead yeasts of K. lactis and C. jadinii cells have comparable positive effects on gut inflammation.
(A-B) Mice received *Kluyveromyces lactis* (K. lac) or *Cyberlindnera jadinii* (C. jad) supernatant (SN) and then dextran sulfate sodium (DSS) for 7 days. Vehicle n = 20, K. lac SN n = 20, C. jad SN n = 18. For statistical comparisons, (*) indicates K. lactis SN versus Vehicle, and ($) indicates *C. jadinii* SN versus Vehicle. (C-D) Mice received *K. lac* or *C. jad* dead bacteria (DE) and then dextran sulfate sodium (DSS) for 7 days. Vehicle n = 6, K. lac DE n = 9, C. jad DE n = 7. For statistical comparisons, (*) indicates K. lactis DE versus Vehicle, and ($) indicates *C. jadinii* DE versus Vehicle. A-C. Weight of DSS-exposed mice. B-D. Disease activity index (DAI) of DSS-exposed mice. *,$p <0.05, **,$$p <0.01, $$$p<0.001.

Supplemental Figure 3

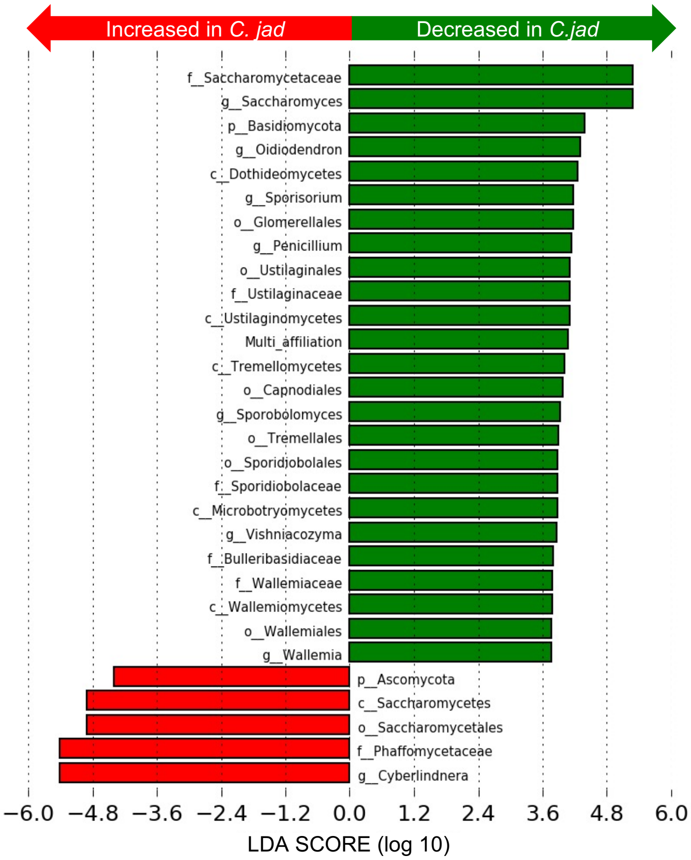

Supplemental Figure 3: *C. jadinii* administration triggers decreases in the abundance of many fungal genera.
Taxa with the largest differences (LDA >2) in abundance by linear discriminant analysis (LEfSe) (LDA >2).
